# Biological Events in Periodontal Ligament and Alveolar Bone Associated with Application of Orthodontic Forces

**DOI:** 10.1155/2015/876509

**Published:** 2015-09-02

**Authors:** L. Feller, R. A. G. Khammissa, I. Schechter, G. Thomadakis, J. Fourie, J. Lemmer

**Affiliations:** ^1^Department of Periodontology and Oral Medicine, Sefako Makgatho Health Sciences University, Pretoria 0204, South Africa; ^2^Schulich Faculty of Chemistry, Technion-Israel Institute of Technology, 32000 Haifa, Israel; ^3^Private Practice, 15 School Road, Morningside, Johannesburg 2057, South Africa

## Abstract

Orthodontic force-induced stresses cause dynamic alterations within the extracellular matrix and within the cytoskeleton of cells in the periodontal ligament and alveolar bone, mediating bone remodelling, ultimately enabling orthodontic tooth movement. In the periodontal ligament and alveolar bone, the mechanically induced tensile strains upregulate the expression of osteogenic genes resulting in bone formation, while mechanically induced compressive strains mediate predominantly catabolic tissue changes and bone resorption. In this review article we summarize some of the currently known biological events occurring in the periodontal ligament and in the alveolar bone in response to application of orthodontic forces and how these facilitate tooth movement.

## 1. Introduction

In response to application of orthodontic forces, the strain-induced mechanical stimulation of the cells and their associated extracellular matrix has the capacity directly to regulate integrin expression, focal adhesion proteins, cytoskeletal organization, cell morphology, cell adhesion to extracellular matrices, cell proliferation, and cell differentiation [1], thus influencing bone modelling.

When the extracellular matrix is stressed, isometric tension develops in the cells within the matrix. This isometric tension is equal in magnitude to the mechanical tensional force exerted upon them by the extracellular matrix, leading to changes in their cellular cytoskeleton and architecture with activation of cellular transcription factors [1, 2]. This in turn influences the expression of genes involved in cell attachment, proliferation, differentiation, and apoptosis. Reciprocally, cells have the capacity to transfer tension generated in the actin cytoskeleton by direct mechanical stimulation to extracellular matrix proteins, influencing the three-dimensional organization of the extracellular matrix and its remodelling [2–4].

In this review article we summarize some of the currently known biological events occurring in the periodontal ligament and in the alveolar bone in response to application of orthodontic forces and how these facilitate tooth movement.

## 2. Aspects of Bone Remodelling Related to Orthodontic Tooth Movement

Bone remodelling or bone turnover is a physiological process comprising osteoclast-mediated bone resorption coupled with osteoblast-mediated bone formation. The bone mass is determined by the balance between resorption and formation of bone. Following bone resorption, biologically active agents including bone morphogenetic proteins (BMPs), fibroblast growth factor (FGF), and transforming growth factor-*β* (TGF-*β*) are released from the organic matrix of the resorbed bone into the local microenvironment, inducing osteoblast-mediated bone formation [5, 6].

Bone-resorbing osteoclasts originate from the monocyte/macrophage lineage of the haematopoietic stem cells in the bone marrow. Osteoclast differentiation is mediated by various cytokines, hormones, and growth factors with the receptor activator of nuclear factor-*κβ*- (RANK-) RANK ligand- (RANKL-) osteoprotegerin (OPG) signalling pathway being an essential regulator. The function of osteoclasts is mediated by complex interactions between several agents including parathyroid hormone, calcitonin, vitamin D, macrophage colony-stimulating factor (MCSF), tumour necrosis factor (TNF), oestrogen, and several interleukins (IL) [5, 7–9].

RANKL, both in a membrane bound form and as a soluble ligand, is expressed by osteoblast precursors and other bone stromal cells. These cells also express OPG, a soluble decoy receptor for RANKL. While RANKL binds to RANK on osteoclast precursors inducing their differentiation, maturation, and activation, OPG indirectly inhibits osteoclastogenesis mediated by RANK-RANKL. Thus, the balance between RANKL and OPG activities on the background of the other biological mediators mentioned above will determine the extent and rate of the bone resorption [5, 7, 8, 10, 11]. In fact, some cytokines such as IL-6 and TNF-*α* have the capacity to induce the expression of RANKL on osteoblasts and T lymphocytes and possibly other periodontal ligament cells, thus stimulating osteoclastogenesis [8].

At sites of bone resorption the mature osteoclasts attach to the mineralized matrix, releasing hydrogen ions from cytoplasmic vesicles. This together with proton pumping mediates the dissolution of inorganic crystalline apatite, and this process is followed by proteolytic enzyme-induced degradation of the inorganic bone matrix [6, 12, 13].

In contrast, osteoblasts originate from progenitor cells within the bone marrow stroma. BMPs, ligands of the Wnt signalling pathway, FGF, IGF, and transforming growth factor- (TGF-) *β* are biological agents mediating proliferation and differentiation of osteoblast precursors, ultimately promoting bone formation [5, 14, 15].

The Wnt/*β*-catenin-dependent canonical signalling pathway plays an important role in bone formation and remodelling. It upregulates the expression of genes which induce the differentiation and maturation of osteoblast precursor cells (osteoblastogenesis) and induces an increase in OPG : RANKL ratio, thus inhibiting osteoclastogenesis [16–20].

**Figure 1:**
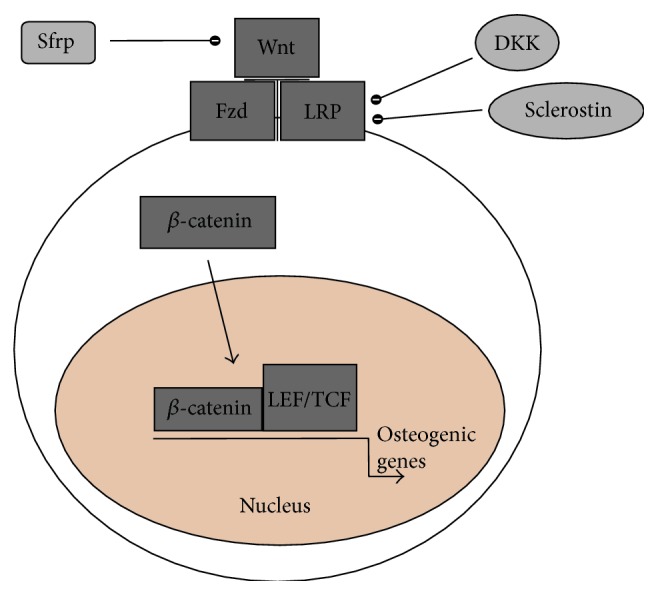
A simplistic illustration of the Wnt/*β*-catenin cellular signalling pathway in an osteoblast. Activation of the Frizzled- (Fzd-) LRP5/LRP6 receptor complex at the plasma membrane by Wnt proteins brings about accumulation of *β*-catenin in the cytoplasm, which then translocates to the nucleus, where it activates the LEF/TCF transcription factors, regulating expression of osteogenic genes; Sfrp suppresses Wnt activation and Dickkopf (Dkk) and sclerostin bind to LRP5 and LRP6 blocking the Wnt signalling [18, 20].

Upon stimulatory activation of Wnt main receptor Frizzled and Wnt coreceptors lipoprotein receptor-related protein 5 (LRP5) and LRP6 that together constitute a ternary complex unit (receptor unit) at the cell membrane [17, 18], there is an accumulation of *β*-catenin in the cytoplasm. This then translocates to the nucleus where it binds and activates the transcription factors lymphoid enhancer factor (LEF) and T-cell factor (TCF), inducing upregulation of expression of Wnt target “osteogenic genes” [17, 18, 20], thus mediating osteoblastogenesis with consequent bone formation (Figure 1). Furthermore, the Wnt/*β*-catenin signalling pathway, through transcriptional suppression of RANKL and upregulation of expression of OPG, has the capacity to regulate osteoclastogenesis [19].

There are several secreted biological agents that can inhibit the Wnt/*β*-catenin signalling pathway. These include the secreted Frizzled-related proteins (Sfrps) that inhibit the interaction between Wnt ligands and Frizzled and the Dickkopf proteins (Dkks) and sclerostin, the latter encoded by the SOST gene that, by interacting with LRP5/LRP6, inhibits the activation of the Wnt/*β*-catenin signalling pathway (Figure 1) [17, 18, 20, 21]. Thus, Wnt/*β*-catenin-mediated bone formation and remodelling are regulated by the balance between a number of agonists and antagonists.

Activation of specific transcription factors, predominantly Runx 2, and activation of both the BMP pathway and the Wnt signalling pathway together mediate the commitment of mesenchymal stem cells towards an osteogenic lineage and thus induce osteoblastogenesis. In turn, mature activated osteoblasts express osteogenic genes encoding several proteins and enzymes including bone sialoprotein, osteocalcin, alkaline phosphatase, and type 1 collagen, all essential for the formation of extracellular organic matrix and for its subsequent mineralization [5, 12, 14, 15, 22].

With time, the layer of newly formed bone becomes thicker and some of the active osteoblasts will become enclosed in lacunae as osteocytes [12, 23]. This process is regulated by parathyroid hormone, vitamin D, and certain growth factors including FGF, PDGF, IGF, and TGF-*β* [5].

In the context of orthodontic tooth movement, in response to mechanical loading of bone, there is upregulation of expression of the canonical Wnt/*β*-catenin signalling pathway with a transcriptional activation of a number of osteogenic genes, promoting the differentiation and maturation of osteoblasts, with bone formation [16]. It is probable that the Wnt/*β*-catenin signalling pathway interacts with other intracellular signalling pathways such as those activated by prostaglandins, nitrous oxide (NO), or BMPs, and these interactions promote bone formation [16]. Furthermore, in the context of orthodontic tooth movement, the applied forces are transmitted* via* the stressed tissue matrix to local cells in the periodontal ligament (PDL) and alveolar bone, stimulating the cells to release proinflammatory, angiogenic, and osteogenic agents [7, 24]. These, in turn, trigger the process of remodelling of the PDL and adjacent alveolar bone [7, 24], ultimately enabling tooth movement.

## 3. Biological Events Occurring in Response to Mechanical Loading

Orthodontic load-induced deformation of the bone matrix generates a complex, nonuniform biophysical environment within the bone consisting of fluid flow, direct mechanical strain, and electrokinetic effects. The latter are local electrical fields (of the order of 6 mV/cm) that occur endogenously owing to piezoelectric effects and/or streaming potentials. These biophysical signals stimulate mechanosensors which in turn activate intracellular signalling pathways in osteocytes contributing to bone-cell responsiveness [25].

Tensile strains in the PDL and alveolar bone have the capacity to stimulate upregulation of expression of osteogenic genes with the differentiation of osteogenic progenitor cells into mature osteoblasts which deposit osteoid that subsequently undergoes mineralization [1, 3, 10]. It appears that tensile stress-induced bone formation is not associated with proliferation of osteoblasts but rather with an increase in the rate of differentiation and maturation of osteoblast precursor cells [13, 22]. Compressive strains in the PDL and alveolar bone, on the other hand, upregulate the expression of RANK either directly or through the action of IL-1*β* and prostaglandins, initiating osteoclast-mediated bone resorption [7, 26].

It appears that, in mesenchymal stem cells in bone, mechanically induced strains activate the regulated protein kinase ERK 1/2, a member molecule of mitogen-activated protein kinase (MAPK) intracellular signalling pathways. In turn, the ERK 1/2 pathway activates the Runx 2 transcription factor, a critical regulator of osteogenic gene expression, bringing about differentiation and maturation of osteogenic precursor cells into osteoblasts that produce alkaline phosphate, collagen type 1, and osteocalcin [22, 27].

Mechanical strains thus have the capacity to activate the ERK 1/2-Runx 2 intracellular pathway in bone mesenchymal stem cells, committing them to an osteogenic lineage and mediating the differentiation of these cells into bone forming osteoblasts [22]. In addition to the ERK 1/2 cascade, the MAPK intracellular signalling pathways also include the c-JUN N-terminal kinase (JNK) cascade and the p38 cascade. In response to mechanical strains, the MAPK pathways, particularly the p38 cascade, upregulate production of inflammatory cytokines and RANKL by osteoblasts, thus initiating osteoclastogenesis and promoting bone remodelling [8]. It appears that the selective activation of the MAPK intracellular signalling pathways,* via* either ERK 1/2, JNK, or p38, depends on the magnitude of the tissue strain [27] that in the context of orthodontic treatment is determined by the characteristic of the applied orthodontic forces.

## 4. The Periodontal Ligament and Orthodontic Tooth Movement

The PDL is a fibrous structure of high tensile strength and relatively slight elasticity, connecting the tooth to the alveolar bone. When load-induced displacement of the interstitial fluid occurs, the PDL fibers will become electrically charged which causes the fibers to be repelled from one another and then they rebound simulating an elastic effect. Colloidal substances in the interstitial fluid act as a damper to the pseudoelastic PDL fibers: the fibers, fluids, and colloids constitute a viscoelastic system. The thixotropic periodontal matrix colloids transit from the “sol” to the “gel” state depending upon the amount of stress to which they are exposed. In this transition the colloidal solution transforms into an integrated polymeric network. This way, the rate of fluid flow through the PDL interstitial spaces is governed [28].

During the initial phase of orthodontic tooth movement in which the tooth is displaced within the PDL space, the PDL becomes hyperaemic, oedematous, and infiltrated with acute inflammatory cells [3, 24, 26]. The increase in inflammatory fluid and cellular infiltrate in the PDL and adjoining alveolar bone affect the viscoelastic properties of both the bone and the ligament. There is a progressive decrease in the tensile strength of the collagen bundles as a result of release of matrix metalloproteinases (MMPs) and other catabolic agents which disrupt the cross-linkages and molecular integrity of the extracellular matrix [12, 29], and the hydrodynamic damping effect of the PDL is decreased, while the elasticity of the bone is increased [26, 29].

Formally, the theoretical model describing the induction of tooth movement after the application of an external force includes several stages: first, strain in the matrix of the PDL and the alveolar bone results in fluid flow alterations in both tissues. This causes cell deformation, which results in activation of fibroblasts and osteoblasts in the PDL and osteocytes within bone. Finally, a combination of remodelling of the PDL and remodelling of the alveolar bone enables the tooth to move [12].

The applied orthodontic forces compress, stretch, or twist the collagen fibers and alter the fluid flow within the periodontal ligament space [12], thus disrupting the configuration of the extracellular matrix proteins, exposing molecules that can activate the fibroblasts* via* integrins and focal adhesion domains. Some of these newly exposed activating molecules and the mechanically induced signals generated by the orthodontic force bring about the expression of genes encoding several proteins and enzymes essential for the remodelling of the extracellular matrix of the PDL. These proteins include collagen and fibronectin, and the enzymes include MMPs, serine proteases, aspartate proteases, and cysteine proteases that degrade and remodel the collagen and other macromolecules [1, 14, 30].

Cells in the PDL react differently to tensile and to compressive strains, mediating predominantly catabolic tissue changes at sites under compression and predominantly anabolic activity at sites under tension [3]. This coordinated remodelling of the PDL is essential for orthodontic tooth movement.

The passage of ions through mechanosensitive ion channels can activate cellular functions in response to applied forces. The size of the channels is regulated by load-induced stretching of the plasma membrane. Tensional forces of sufficient magnitude will stretch the plasma membrane increasing the channel diameter and consequently the flow of ions so that the electrical conductivity of the membrane is increased. This activates intracellular signalling eliciting cellular responses [31].

## 5. Alveolar Bone and Orthodontic Tooth Movement

If the compressive strains generated by orthodontic forces exceed the elastic limit of bone, either microfractures or degenerative changes will occur [12]. This will disturb the local osteocytes in their lacunae and their cytoplasmic processes in the canaliculi interconnecting the lacunae within the substance of the bone [32, 33]. Within this three-dimensional lacunocanalicular network, osteocytes communicate with one another and with osteoblasts through gap junctions at the extremities of their cytoplasmic processes [3, 12, 32].

**Figure 2:**
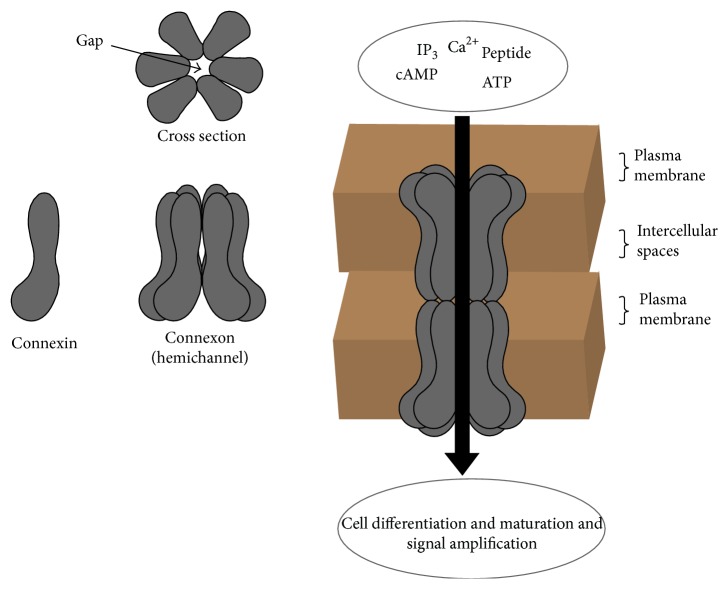
Six connexin molecules constitute a connexon hemichannel in the plasma membrane. Two hemichannels of adjacent cells docked together form a functional gap junction through which small molecules and peptides mediating cell proliferation and differentiation and maturation and amplification of intercellular signals can pass.

A gap junction is a channel connecting the cytoplasm of two adjacent cells, which allows the passage of ions, metabolites, and small signalling molecules such as ATP and Ca^2+^ [34]. The channel is composed of two hemichannels, each hemichannel belonging to one of the adjoining cells. Each hemichannel is termed a connexon and this is an assembly of 6 connexin proteins (Figure 2). When two connexons, one belonging to the cell membrane of each of the adjacent cells, become opposed and docked in the intercellular space, they form a functional gap junction channel. Electric, chemical, and mechanical factors influence the opening or closing of the gap junction gate. In general, gap junction channels are more often gated open than closed (Figure 2) [35, 36].

Although osteogenic cells express several types of connexins (Cx), Cx 43 is the most common gap junction protein. If the expression of Cx 43 by osteoblasts and osteocytes becomes dysregulated the gap junctions will be functionally deficient. It has been shown that biomechanical stimuli as from application of orthodontic forces can upregulate the expression of Cx 43 enhancing the function of gap junctional communication between bone cells. The gap junctional communication provided by Cx 43 is essential for osteoblast differentiation, maturation, and function and hence for bone remodelling [25].

In addition to Cx 43 gap junction channels, osteoblasts and osteocytes also have in their cell membranes hemichannels (connexons) that are not part of the intercellular gap junctions but that face and are in communication with the extracellular microenvironment [34, 37]. These hemichannels are located in the plasma membrane of the cell body, rather than in the plasma membrane of the dendritic processes [34], and function independently of the Cx 43 gap junction channels [37].

Mechanical loads as from orthodontic forces activate the *α*5*β*1 integrins which mediate opening of Cx 43 hemichannels in osteocytes with the release into the microenvironment of prostaglandin E_2_ and ATP [34, 37, 38]. The same orthodontic forces, through compression and stretching of the periodontal tissues, induce fluid flow shear stresses with the expression of Cox-2 and of prostaglandin E_2_ receptor EP_2_ in osteocytes. Consequent to the activation of EP_2_ receptors by prostaglandin E_2_ there is an increase in Cx 43 expression leading to the assembly of additional functional gap junction channels with improvement in the passage of signalling molecules, enhancing communication between bone cells [39].

Within the lacunocanalicular network, the osteocytes and their cytoplasmic processes are bathed in a fluid that transports signalling molecules, nutrients, and waste products [23, 32, 33, 40–42] and differs significantly from the extravascular interstitial fluid in terms of ionic content [23]. Orthodontic loading or overloading causes fluctuations in the ebb and flow of this lacunocanalicular fluid, with corresponding alterations in the microenvironment of and in the fluid pressure on all bone cells, but particularly on osteocytes confined as they are to their bony location [3, 12, 32, 39, 40]. The viscosity and the biochemical composition of the fluid, the nature of the organic lining, and the physical characteristics of the walls of the lacunocanalicular system all affect the characteristics of the fluid flow [23]. The osteocytes respond to the physical stimulus of fluid flow by generating, amplifying, and transmitting signals through gap junctions, and this cross talk between the osteocytes orchestrates bone remodelling [25, 39, 43].

Orthodontic loading and overloading can cause microdamage to the bone, and the associated alterations in fluid flow in the lacunocanalicular network can also injure osteocytes and induce their apoptosis [12, 33, 43, 44]. Signals from osteocytes in the process of apoptosis have the capacity to recruit osteoclasts to the zone of the microdamage, where, together with the other bone cells, they participate in bone remodelling [45]. Consequent to bone damage, local release of inflammatory mediators, cytokines, and growth factors such as NO, endothelins, prostaglandin E_2_, vascular endothelial growth factor (VEGF), and TGF-*β* further promote bone remodelling facilitating orthodontic tooth movement [13, 46].

Orthodontic forces can also cause periodontal tissue compression with diminishing blood flow and the potential for ischaemic alveolar bone necrosis. Mild ischaemia and hypoxia will cause cells in the local microenvironment to express specific genes that regulate metabolic processes allowing them to adapt to the altered microenvironment [1, 47]. Under these circumstances there is release of growth factors and other biological agents from the bone matrix and from the compressed blood vessels, with the recruitment of local bone progenitor cells and subsequent bone remodelling [1, 13, 45, 46, 48, 49].

If the orthodontic forces are within the accepted therapeutic range, the compressive ischaemic bone necrosis will be insignificant, resorptive remodelling will occur, and controlled tooth movement will follow [1, 13, 46]. On the other hand, if excessive orthodontic forces exceed the adaptive capacity of the affected tissues, the compressive forces may cause cell death, tissue hyalinization in the PDL, a zone of alveolar bone necrosis, and external root resorption. The hyalinized tissue becomes degraded and resorbed, and the damaged alveolar bone adjacent to the hyalinized tissue undergoes undermining resorption, with some degree of external root resorption [1]. Subsequently, neovascularization and regeneration of the damaged PDL and alveolar bone occur [1, 13, 46].

## 6. Conclusion

Applied orthodontic forces are transmitted* via* the stressed tissue matrix to local cells in the periodontal ligament and alveolar bone, stimulating the cells to release proinflammatory, angiogenic, and osteogenic agents. These trigger the process of remodelling of the periodontal ligament and adjacent alveolar bone.

In bone, mechanically load-induced strains in the cells and in their extracellular matrix and stresses of fluid flow can mediate changes in gene expression of cells. This culminates in initiation of osteoclastogenesis and in differentiation of osteogenic cells with consequent production of nitric oxide, prostaglandins, osteocalcin, osteopontin, alkaline phosphatase, and type 1 collagen, thus promoting bone formation and remodelling.
